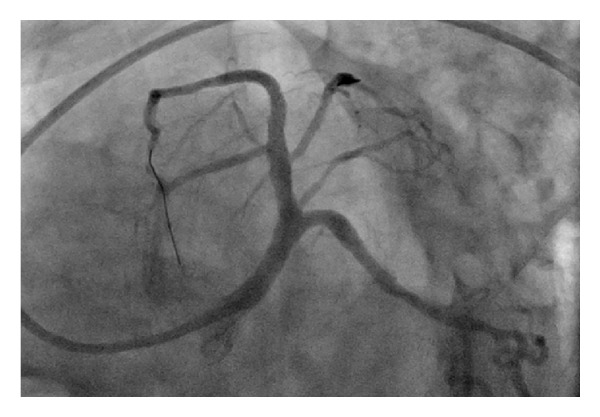# ST-Elevation Myocardial Infarction after Pharmacologic Persantine Stress Test in a Patient with Wellens' Syndrome

**DOI:** 10.1155/2014/530451

**Published:** 2014-04-02

**Authors:** Kunal Patel, Fady Alattar, Jayanth Koneru, Fayez Shamoon

**Affiliations:** Department of Cardiovascular Services, Trinitas Regional Medical Center, Seton Hall University, 225 Williamson Street, Elizabeth, NJ 07202, USA

## Abstract

Wellens' syndrome, also known as LAD coronary T-wave inversion syndrome, is a characteristic ECG pattern that highly suggests critical stenosis of the proximal left anterior descending (LAD) coronary artery. 75% of patients with this finding go on to develop acute anterior wall myocardial infarction within one week unless prevented by early intervention on the culprit lesion. Most instances of ST-elevation occurring during cardiac stress testing have been observed with exercise, with only seven cases reported in the literature with pharmacologic stress. We present a case of a patient with no known cardiac disease who presented with chest pain and an ECG consistent with Wellens' syndrome that developed an acute anterior wall ST-elevation myocardial infarction after pharmacologic stress test.

## 1. Introduction

Wellens' syndrome typically presents with the characteristic ECG findings of biphasic T-waves or deep symmetrical T-wave inversions in the precordial leads (leads V1–V4). This ECG finding usually occurs during a pain-free period and is highly suggestive of critical proximal LAD coronary artery stenosis. Given the significant number of patients that will go on to develop acute anterior wall myocardial infarctions, it is critical that all physicians recognize this classic ECG pattern and institute measures for the patient to undergo urgent coronary angiography and revascularization. If left untreated, managed medically, or further risk-stratified by cardiac stress testing, the patient may develop an extensive myocardial infarction or sudden death.

## 2. Case Presentation

A 72-year-old Hispanic male with a past medical history of hypertension, hyperlipidemia, end-stage kidney disease on hemodialysis, and cerebrovascular accident 5 years before presented to the emergency department with midsternal crushing chest pain that awoke him up from sleep at 7 am in the morning. The pain radiated to his jaws bilaterally and was associated with diaphoresis. The pain continued for 30 minutes until EMS arrived and improved after sublingual nitroglycerin and chewable aspirin 325 mg. Significant social history included a history of cigarette smoking. No additional pertinent history was appreciated including review of systems, family history, and social history. The patient's blood pressure on presentation was 137/70 mmHg, heart rate was 102 bpm, and physical exam was documented as essentially within normal limits.

**Figure 1:**
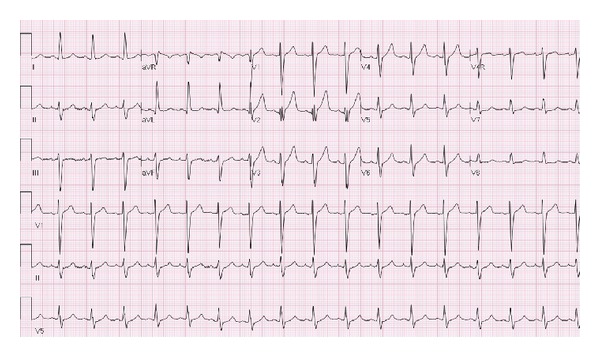


The patient's electrocardiogram upon presentation (Figure 1) showed a sinus tachycardia at a rate of 102 beats per minute, LVH with QRS widening, and prolonged QT interval. Basic laboratory tests including complete blood count and metabolic profile were unremarkable. Initial cardiac markers (CK, CK-MB, BNP, and troponin) were also within normal limits. Thereafter, the patient was admitted to the telemetry floor and started on aspirin and Plavix antiplatelet therapy, statin, beta blocker, and ARB.

**Figure 2:**
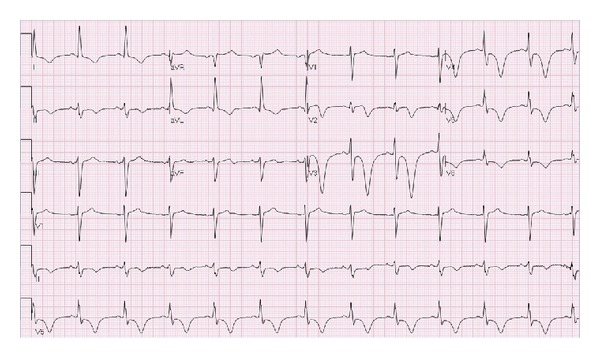


Throughout the hospitalization the patient remained chest-pain-free and subsequent cardiac markers remained normal. The patient was scheduled to undergo pharmacologic dipyridamole (Persantine) stress testing with nuclear imaging for further risk stratification. A repeat ECG taken before the test is displayed in Figure 2. It showed a normal sinus rhythm at 74 bpm, LVH with QRS widening, 1 mm ST-segment depressions in leads V3–V6, and diffuse symmetric T-wave inversions in leads V2–V6, I, and II. Despite these ECG changes, the patient remained asymptomatic and the stress test was performed. At peak dipyridamole stress (55 mg intravenously over 4 minutes), the patient reported mild chest discomfort and headache. There were no associated ECG changes. These symptoms resolved after administration of IV aminophylline 125 mg given during the recovery period.

**Figure 3:**
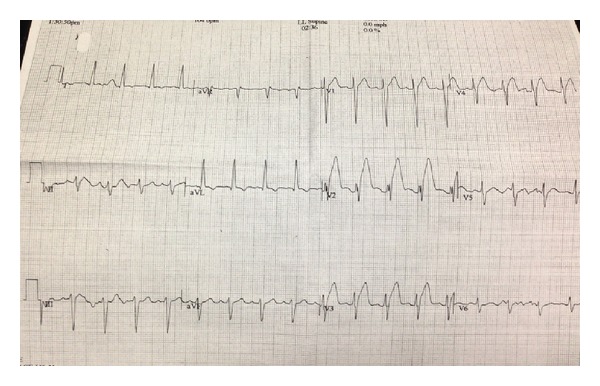


**Figure 4:**
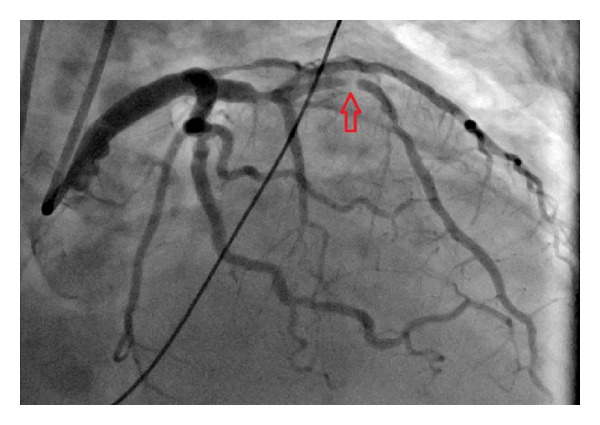


**Figure 5:**
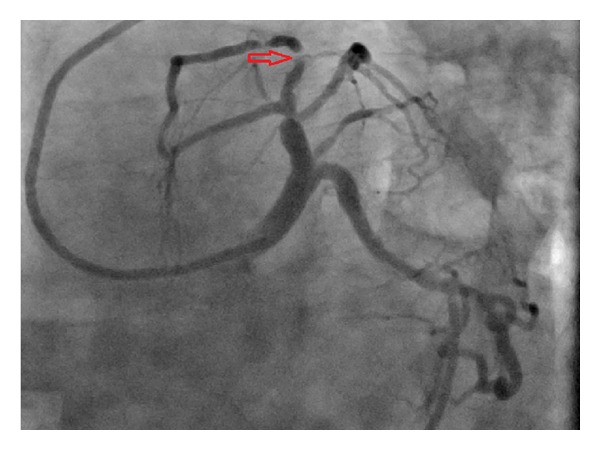


**Figure 6:**
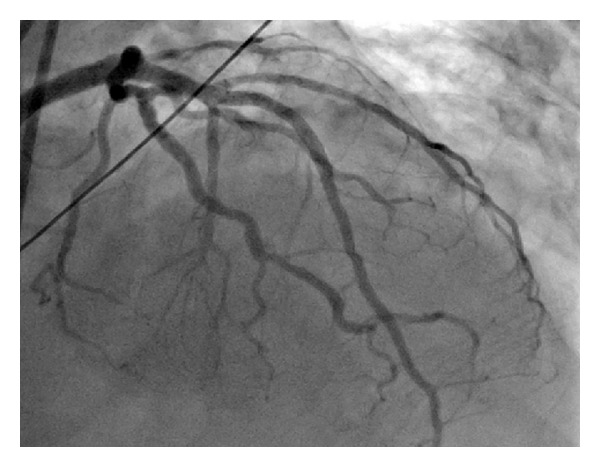


Roughly sixty minutes after completion of the stress test the patient experienced severe retrosternal chest pain and diaphoresis. A subsequent electrocardiogram revealed sinus tachycardia at 102 bpm (Figure 3) and ST-elevations in leads V1–V4 consistent with an acute anterior wall ST-elevation myocardial infarction. The patient was taken to the cardiac catheterization lab where a coronary angiogram (Figures 4 and 5) revealed a diffusely diseased LAD with a 99% obstructive lesion in the mid-LAD. Percutaneous transluminal coronary angioplasty (PTCA) was performed and two drug-eluting stents were placed in the mid-LAD, after which the flow reverted back to normal (Figures 6 and 7). The patient recovered uneventfully and was advised to continue antiplatelets, beta blocker, and statin upon discharge. He was also counseled against the use of tobacco.

## 3. Discussion

A subgroup of unstable angina with high risk of progression to anterior wall myocardial infarction has been described early in the literature as “left anterior descending coronary T-wave syndrome” [1]. In early 1980s, de Zwaan, Wellens, and others documented the subtle ST segment changes with characteristic T-wave pattern in those patients [2].

ECG patterns of Wellens' syndrome can be divided into variants. The larger group of patients (including our patient) demonstrate deep symmetrical T-wave inversion that can extend from V1 to V5. The other variant (24% of cases) [3] consists of biphasic T-waves in leads V2 and V3 with isoelectric or minimally elevated ST segment. Acute U wave inversion was also reported in patients with unstable angina due to critical LAD lesions, but some authors believe it represented merely the terminal part of biphasic T-wave inversion [4, 5]. The lesion is obstructive (diameter stenosis > 50%) in all patients in Wellens' series [1]. It involves either the proximal or the middle LAD artery. The sensitivity, specificity, and positive predictive value of T-wave inversion in the precordial leads for significant LAD stenosis are 69%, 89%, and 86%, respectively [6].

Our case highlights several concerning features of Wellens' syndrome. At first the ECG changes developed during pain-free period. In Wellens' study, the characteristic pattern was seen in the first 24 hours in 60% of cases only (108 patients out of 180) [1, 5].

Despite the ominous ECG changes, our patient had limited myocardial injury as reflected by normal troponin levels. Baseline echocardiography and gated rest images were also normal. In the original series of patients, Wellens reported myocardial damage in 12% of cases only [1]. Actually, preserved precordial, R wave progression is considered as a criterion of Wellens' sign [7]. We came across one reported case of exercise SPECT myocardial perfusion scan done in a patient with Wellens' syndrome. The rest images showed a small anteroseptal defect at rest with normal motion on echocardiography. The defect has been attributed by the authors to breast attenuation artifact or hibernating myocardium [8]. The later observations may point to the possibility that the characteristic T-wave changes happen as a sequel to reperfusion after transient ischemic event [9].

Wellens' syndrome can progress to myocardial infarction over a course of days to weeks. STEMI has been precipitated in cases of Wellens' syndrome after regular exercise stress [8]. Here, the remarkable aspect of this case is that progression to an acute STEMI happened after a pharmacologic based stress test using a vasodilator drug (Persantine). This could be explained by “coronary steal phenomenon” that develops when a vasodilator drug leads to decrease in the collateral flow to ischemic areas in patients with multivessel disease. Using PET (positron emission tomography) perfusion scanning, van Tosh et al. were able to demonstrate that coronary steal developed between 12 to 39% of patients with CAD during pharmacologic vasodilator stress test [9].

Recognition of Wellens' syndrome and appropriate intervention prevent a potentially devastating myocardial infarction. It is prudent to recommend coronary angiography as the initial diagnostic modality in patients with ECG suspicious of Wellens' syndrome as stress tests including pharmacologic ones carry a risk of progression to myocardial infarction.

## Figures and Tables

**Figure 7:**